# On the Organic Diseases and Functional Disorders of the Stomach

**Published:** 1856-06

**Authors:** 


ART. VI.—*On the Organic Diseases and Functional Disorders of the Stomach.* By GEORGE BUDD, M. D., F. R. S., Professor of Medicine in King's College, London; late Fellow of Caius College, Cambridge; Author of a "Treatise on Diseases of the Liver," etc., pp. 250, 8vo. Philadelphia: BLANCHARD AND LEA. 1856. New York: S. S. & W. WOOD. 1856.

We are indebted to two publishing houses for copies of the work whose title is given above. Its subject is an important one, inasmuch as the stomach is the principal *entrépot* of the system for food, and is consequently the starting point for many disorders. Modern philosophy has, however, considerably subtracted from the mass of duty formerly assigned to the stomach. When it was considered as the sole digestive organ, acting alike on all forms of ingesta, its gastric juice forming a universal solvent, it well deserved those extravagant ideas of its importance which were inculcated by all our authors.

But when we come to an understanding of the real function of the stomach, and regard it as a simple enlargement of the alimentary tube, possessing mucous membrane not widely different from that of other portions of the same tube, and secreting a fluid which leaves the great mass of our food untouched or unchanged, except as to the single quality of fluidity, we are disposed to go too far the other way, and look on the stomach as a much less efficient and important organ than it is.

The recent changes in the accredited physiological doctrines concerning the function of the stomach require a revision of its diseases, and more especially its functional disorders. Very many of our theories of disease and of diet are incompatible with our theory of health, and a most inviting field is here opened for the practical physician.

We are disappointed to find that Dr. Budd has not addressed himself to this task. His ability as a teacher, and his practical tact in disease would have well qualified him for such a labor. But nowhere in his pages do we find more than an incidental mention of the healthy functions of the stomach, and nowhere is its philosophy brought to illustrate its diseases. Neither do those recent investigators—Bernard and others—who have done so much for this department of science receive one word of mention. The existence of their discoveries or of themselves could not be inferred from any thing within the covers of "Budd on the Stomach."

We regard this as a great fault in the work under notice, but are willing to leave it with this bare mention, and turn from the negative to the positive qualities of the book. Wanting in what we deem an essential feature of such a book, it has still many merits to arrest the eye and to commend it to the practical man.

The book is divided into sixteen lectures, the first of which is devoted to "self-digestion of the stomach," a subject of considerable interest with reference to post-mortem appearances. In the phrase "self-digestion" the author includes, not only softening after death, from the action of the gastric juice, but cases of perforation and complete solution of the great *cul-de-sac*, and the lower portion of the œsophagus. Sometimes the perforation is carried into the pleural cavity, and the author quotes a case occurring to Sir Astley Cooper, in which "œsophagus was dissolved, and bread and cheese were found extravasated in the chest." This, of course, only occurs when the stomach is engaged in the process of natural digestion at the time of death, so that it will be found in persons dying soon after a meal, and should be taken into account in recording post-mortem appearances.

Dr. Marshall Hall once mentioned to us, that in an autopsy made by him on a child dead some three days, he found that the gastric juice had corroded the œsophagus, and all the contents of the posterior mediastinum, *with the exception of the nerves*, which were not acted on by the fluid. Thus nature had effected for the great physiologist an accurate dissection of the vagus and splanchnic nerves, with the bronchial and œsophageal plexus. He mentioned it to us as a hint for conducting nervous dissections. By the use of rennet this process might be artificially imitated. A temperature near that of the body would be necessary to success.

We find (on page 141) it is stated that the act of vomiting of itself excites a secretion of gastric juice. It is well known that symptoms of gastric disturbance are a common accompaniment of phthisis, and M. Louis refers the softening of the stomach often found after death, in consumptive patients, to this cause, considering the gastric disease a part of the general condition. To this Dr. Budd objects that softening, and even perforation, is found in phthisical patients who have suffered little from gastric disturbance, while again in patients who have suffered very much from it, very little change is found in the stomach after death. Dr. Budd, therefore, looks on the softening as a purely post-mortem appearance, and "that the gastric symptoms in such cases originate, not in structural disease of the stomach, but in undue and untimely secretion of gastric juice, and other functional disorder, excited by reflex nervous influence." (From the lung, of course, implied.)

The chapter on sympathetic disorders of the stomach is especially valuable to the practitioner, and abounds in practical suggestions.

It is not our purpose to devote much of our at present crowded space to the further review of Dr. Budd's volume. Reiterating that it is deficient in a most important particular, (that of views drawn from an enlightened physiology) it is still a work which will prove of value to the practitioner—for whom, we suppose, it was especially intended. Its scope of subjects is as large as is warranted by our present capacities for diagnosis, or our knowledge of pathology. It makes no unnecessary divisions of the diseases of the organ, nor does it wander from it.

For sale by PHINNEY & Co.

